# Total Fume and Metal Concentrations during Welding in Selected Factories in Jeddah, Saudi Arabia

**DOI:** 10.3390/ijerph7072978

**Published:** 2010-07-22

**Authors:** Mansour Ahmed Balkhyour, Mohammad Khalid Goknil

**Affiliations:** Environmental Sciences Department, Faculty of Meteorology, Environment and Arid Land Agriculture, King Abdulaziz University, P.O. Box 80208, Jeddah 21589, Saudi Arabia; E-Mail: mbalkyhour@kau.edu.sa

**Keywords:** fume, particulates, occupational exposure

## Abstract

Welding is a major industrial process used for joining metals. Occupational exposure to welding fumes is a serious occupational health problem all over the world. The degree of risk to welder’s health from fumes depends on composition, concentration, and the length of exposure. The aim of this study was to investigate workers’ welding fume exposure levels in some industries in Jeddah, Saudi Arabia. In each factory, the air in the breathing zone within 0.5 m from welders was sampled during 8-hour shifts. Total particulates, manganese, copper, and molybdenum concentrations of welding fumes were determined. Mean values of eight-hour average particulate concentrations measured during welding at the welders breathing zone were 6.3 mg/m^3^ (Factory 1), 5.3 mg/m^3^ (Factory 2), 11.3 mg/m^3^ (Factory 3), 6.8 mg/m^3^ (Factory 4), 4.7 mg/m^3^ (Factory 5), and 3.0 mg/m^3^ (Factory 6). Mean values of airborne manganese, copper, and molybdenum levels measured during welding were in the range of 0.010 mg/m^3^–0.477 mg/m^3^, 0.001 mg/m^3^–0.080 mg/m^3^ and 0.001 mg/m^3^–0.058 mg/m^3^ respectively. Mean values of calculated equivalent exposure values were: 1.50 (Factory 1), 1.56 (Factory 2), 5.14 (Factory 3), 2.21 (Factory 4), 2.89 (Factory 5), and 1.20 (Factory 6). The welders in factories 1, 2, 3, and 4 were exposed to welding fume concentration above the SASO limit value, which may increase the risk of respiratory health problems.

## 1. Introduction

Occupational exposure to welding fume is a serious occupational health problem all over the world. Welding is a major industrial process used for joining metals. One of the most common types of welding technology is manual metal arc welding (MMAW) which uses a flux coated electrode. In this type of welding process, high heat from an electric arc is used to melt and fuse the metal at the joint between the two parts. All welding processes produce fumes, but maximum fumes are produced during arc welding. In MMAW, fume arises by vaporization of the core metal and flux components of the electrode. Vaporized metals react with air, producing metal oxides which condense and form fumes consisting of particles [1–3].

Welding fumes are a complex mixture composed of different metals. The composition and the rate of generation of welding fumes depend on the components of the metal being welded, coatings, types of electrodes or filler materials, operating conditions of the welding process (temperature, current), and the technique and skill of the welder. Types of metals commonly found in welding fumes include aluminum, beryllium, cadmium oxides, chromium, copper, fluorides, iron oxide, lead, manganese, molybdenum, nickel, vanadium and zinc oxides. Fumes from mild steel welding contain mostly iron, along with small amounts of additive metals like manganese, copper, and molybdenum [4–6].

Most of the particles in welding fume are less than 1μm in diameter when produced, but they appear to grow in size with time due to agglomeration [1,3,6,7]. Particle (0.5–0.7 μm in diameter) concentrations of welding fumes were reported to range between 9,800 particles/cm^3^ and 82,000 particles/cm^3^, depending on voltage level [8]. The average particle size distribution of flux cored arc welding fumes by percentage was reported as: aerodynamic diameter >5.8 μm: 6.01%, 1.1–5.8μm: 3.04%, 0.7–1.1 μm: 19.9%, 0.4–0.7 μm: 23.3%, and <0.4 μm: 47.7% [9].

Exposure to welding fumes can cause numerous health problems. The degree of the risk to welder’s health from fume exposure depends on the composition, concentration, and the length of time the welder is exposed. When inhaled, welding fumes can enter the lungs, bloodstream, brain nerve cells, spinal cord and other organs and can cause both short and long term health effects. Acute exposure to welding fumes can result eye, nose, and throat irritation, fever, chills, nausea, shortness of breath, muscle pain and metallic taste in the mouth. Chronic exposure to welding fumes can result in respiratory effects, including coughing, decreased pulmonary function, and occupational asthma [10–15].

Many of the reported epidemiology studies are difficult to compare because of the wide differences in worker populations, industrial settings, welding techniques, duration of exposure, and other occupational exposures besides welding fumes. Epidemiological studies have indicated that large numbers of welders experience some type of respiratory illness. Respiratory effects observed include acute and chronic bronchitis, air passage irritation, pneumoconiosis “arc welder’s siderosis”, occupational asthma, and possible increase in the incidence of lung cancer [10–12,15–22].

The presence of soluble metals such as iron, chromium, nickel, copper, and manganese, and the various complexes formed by these different metals in welding fumes are likely to be important in the pulmonary responses observed after welding fume exposure [23]. Manganese is known to be bio-available to and neurotoxic for the central nervous system. Although an essential metal, manganese overexposure may cause manganism, a Parkinson-like syndrome [24], although epidemiological evidence linking welding exposures to Parkinson’s disease is still controversial [25].

The aim of this study was to investigate workers welding fume exposure levels in some industries in Jeddah, Saudi Arabia.

## 2. Experimental Section

The investigation was carried out in welding areas of six factories located in Jeddah. These factories were selected to be representative of manual metal arc welding processes. These factories were Al Kawther, Al Mutlak, Jamjoom, Binladin, Hidada (plant 1), and Hidada (plant 2). Factories were represented by numbers in the results in mixed order. Sizes of the work places, number of total workers and welders, work schedule and electrode types during the welding are presented for each factory in Table 1.

Ten samples were collected from each factory for determination of particulate concentrations in the welding areas. A RAC battery-powered particulate/gas sampler was used for sampling. It consists of vacuum pump (19 lpm), motor (6,000 rpm), flow-meter (0–6 lpm), battery (12V, 4 amp), and battery charger. A 47 mm Millipore filter holder was connected to RAC sampler by plastic tubing. Type AA, 0.8 μm pore size, 47 mm MF-Millipore membrane filters were used in filter holder for particulate collection.

In each factory, the air in the breathing zone within 0.5 m from exposed workers was sampled during an 8-hour shift. One to four samples per shift were collected, each over a period of about two hours. The measured ratio of sampling period to welding period was constant for 91% of samples, so measured concentrations represent the exposure concentrations during work hours.

Samples were collected by drawing air at constant flow rate through a pre-weighed Millipore filter. The filters were weighed using an analytical balance with a minimum measurable weight of 0.1 mg. One to four samples per shift were collected, each over a period of about two hours. The rate of air intake through the filter was adjusted between 2 and 3 liters per minute before the start of sampling. At the end of the sampling period, each filter was carefully transferred from the filter holder into a clean, labeled, and self-sealing plastic box. After sampling, the filters were transferred to the laboratory and left for 24 hours, and weighed to determine the total mass of particulate collected on each filter.

After determining the total welding fume weight on the filter, a metal specific analysis was performed on the filter samples. In the laboratory, the filters were transferred to a 50 mL acid washed beaker using plastic forceps. The samples were digested at 100 °C with addition of 10 mL 70% nitric acid. In order to reduce volatilization watch glasses were placed over the beakers. After 60 minutes, the watch glasses were removed and the samples heated to reduce the volume of acid to near dryness. When cool, distilled water was added and the samples were filtered through a Whatman No.41 filter paper. Manganese, copper, and molybdenum concentrations of particulates were then determined by atomic absorption spectrophotometry.

## 3. Results and Discussion

Materials used in welding were mild steel and alloy of iron. Work places were semi-open. A helmet and face shield were used as protective equipment by the welders, who were not using a mask, except in Factory No 2. Mechanical ventilation was used only in Factory No 2. Natural air movement was occurring in all factories.

Ranges of measured particulate concentrations in the breathing zone of the welders for each factory were: 4.0 mg/m^3^–18.0 mg/m^3^ (Factory 1), 4.5 mg/m^3^–14.0 mg/m^3^ (Factory 2), 6.5 mg/m^3^–57.5 mg/m^3^ (Factory 3), 5.5 mg/m^3^–13.5 mg/m^3^ (Factory 4), 1.0 mg/m^3^–14.0 mg/m^3^ (Factory 5), and 1.5 mg/m^3^–5.0 mg/m^3^ (Factory 6). Higher concentrations were measured when either two welders worked for the same job or welders positioned downwind.

SASO (Saudi Standards, Metrology and Quality Organization) has established permissible exposure levels (PELs) for toxic and hazardous substances. PELs represent 8-hour time weighted average (TWA) concentrations for an 8-hour work shift of a 40-hour work week. SASO was set PELs of 15 mg/m^3^ (total) and 5 mg/m^3^ (respirable fraction) as 8-hour TWA for inert or nuisance dust [26].

Measured particulate concentrations were converted into 8-hour average concentrations in order to evaluate health risks. Break times were assumed as non exposure periods. Table 2 shows calculated 8-hour average particulate concentrations in the welding fumes for each factory. Mean values were 6.3 mg/m^3^ (Factory 1), 5.3 mg/m^3^ (Factory 2), 11.3 mg/m^3^ (Factory 3), 6.8 mg/m^3^ (Factory 4), 4.7 mg/m^3^ (Factory 5), and 3.0 mg/m^3^ (Factory 6).

Exposures to welding fumes in factories 1, 2, 3, and 4 were above the permissible exposure limit value (5 mg/m^3^) established by SASO. Exposures to welding fumes in factories 5 and 6 were below the limit value. Compliance with 8-hour TLV for each factory is determined by t-test. Mean exposures were above the TLV in Factory 1 (75% confidence), Factory 2 (55% confidence), Factory 3 (97.5% confidence), and Factory 4 (97.5% confidence). Mean exposures were below the TLV in Factory 5 (55% confidence) and Factory 6 (99.99% confidence).

Measured average concentration of welding fumes in the breathing zone of welders in a big production plant located in Arak, Iran was 7.9 mg/m^3^ for manual metal arc welding. Welders in this study showed decreased pulmonary function and an increased prevalence in respiratory symptoms [12]. Welding area of four factories in New Zealand mean exposure values of total fume ranged between 0.125 mg/m^3^ and 5.28 mg/m^3^ [14]. Time weighted average concentrations of the welding fumes released during flux cored arc welding at the workers’ breathing zone in six industrial plants were 0.2 mg/m^3^–24.3 mg/m^3^ [27]. Average total fume concentrations ranged from 4.73 mg/m^3^–5.90 mg/m^3^ at the welders’ breathing zone, in a study of professional welders performing shielded metal arc welding on carbon steel under field conditions [28].

Investigations of the respiratory effects of welding exposure in manual arc welders exposed welding processes without respiratory protection reported that respiratory symptoms and chronic bronchitis were more prevalent in welders than in the unexposed control group [16–18,29]. A study comparing the neuropsychological function, emotional status, visual function, and illness prevalence of welders primarily involved in steel welding, and exposed to welding fumes for an average of 24.9 years with unexposed, non welder controls concluded that welders had poorer color vision and emotional status, and increased prevalence of illness and psychiatric symptoms [30].

In our study the welders in factories 1, 2, 3, and 4 were exposed to welding fume concentration above the limit value, which increases the risk of respiratory health problems.

Eight hour average concentrations of measured manganese, copper, and molybdenum in welding fume for each factory are presented in Table 3. SASO has set the following permissible exposure levels (PELs): copper fumes, 0.1 mg/m^3^; manganese, 5 mg/m^3^ (ceiling); molybdenum, 5 mg/m^3^ (soluble), 15 mg/m^3^ (insoluble), as TWA for a normal 8-hour workday and a 40-hour work week [26].

ACGIH has assigned threshold limit values (TLV): Copper fume, 0.2 mg/m^3^; manganese, 0.2 mg/m^3^; and molybdenum 5 mg/m^3^ (soluble), 10 mg/m^3^ (insoluble), as TWA for a normal 8-hour workday and a 40-hour work week [31].

Measured manganese concentrations in all factories were well below the SASO permissible exposure level (PEL 8h-TWA, ceiling) of 5 mg/m^3^. Measured manganese concentrations in factories 1, 2, 4, and 6 were below the ACGIH threshold limit value (TLV 8h-TWA) of 0.2 mg/m^3^, but they were exceeding the limit value in factories 3 and 5 for two samples.

Measured copper and molybdenum concentrations in all factories were well below the SASO permissible exposure levels (PEL 8h-TWA) and ACGIH threshold limit values (TLV 8h-TWA).

Mean values of airborne manganese levels measured during welding were 0.22 mg/m^3^ in Factory 3 and 0.21 mg/m^3^ in Factory 5, which slightly exceed ACGIH threshold limit value (TLV 8h-TWA) of 0.2 mg/m^3^.

Average concentration of manganese in welding fumes in the welders breathing zone was reported as 0.18 mg/m^3^ in an automobile parts manufacturing factory at Tehran, Iran and 1.45 mg/m^3^ (geometric mean) at a Beijing (China) vehicle factory [3,5]. In a study for professional welders performing shielded metal arc welding on carbon steel under field conditions, average concentration of manganese in welding fumes in the welders breathing zone ranged from 0.06 mg/m^3^–0.16 mg/m^3^ [28]. In a study on confined space welding in the construction of a new span of the San Francisco-Oakland Bay Bridge, the mean time weighted average of manganese in air ranged from 0.11–0.46 mg/m^3^ (55% > 0.20 mg/m^3^) [32]. The investigation, after examining several large data sets to characterize manganese, iron, and total particulate mass exposures resulting from welding operations suggested that exposures to manganese are frequently at or above the current ACGIH threshold limit value of 0.2 mg/m^3^ [33].

The equivalent exposure for a fume composed of several substances is calculated by using addition formula which requires the sum of the individual concentration (C)/TLV ratios [1,26]:

$${\text{E}}_{\text{m}}={\text{C}}_{1}/{\text{TLV}}_{1}+{\text{C}}_{2}/{\text{TLV}}_{2}+{\text{C}}_{3}/{\text{TLV}}_{3}+\ldots \ldots +{\text{C}}_{\text{n}}/{\text{TLV}}_{\text{n}}+{\text{C}}_{\text{rest}}/{\text{TLV}}_{\text{rest}}$$

where 1, 2,… n represent the individual substances and “rest” indicates that fraction of the fumes for which either there is no analysis or no TLV, and which is assigned to typically have a TLV_rest_ corresponding to that for respirable fraction of nuisance dust (5 mg/m^3^). E_m_ is the equivalent exposure for the mixture. The value of E_m_ will not exceed the unity for satisfaction of the TLV.

Ranges of calculated equivalent exposure values were: 1.19–1.70 (Factory 1), 1.03–2.31 (Factory 2), 3.53–7.13 (Factory 3), 2.05–2.46 (Factory 4), 1.23–3.96 (Factory 5), and 0.95–1.40 (Factory 6). Mean values of equivalent exposures were: 1.50 (Factory 1), 1.56 (Factory 2), 5.14 (Factory 3), 2.21 (Factory 4), 2.89 (Factory 5), and 1.20 (Factory 6). In all factories equivalent exposure levels were exceeding the threshold limit values, except for one sample from Factory 6.

Depending on the welding process and the composition of the welding electrode, manganese may be present in different oxidation states and have different solubility properties. These differences may affect the biological responses to manganese after the inhalation of welding fumes. Manganese intoxication and the associated neurological symptoms have been reported in individual cases of welders who have been exposed to high concentrations of manganese-containing welding fumes due to work in poorly ventilated areas [4]. Long term, low level exposure to welding fumes increases serum concentrations of manganese and iron. Occupational exposure to welding fume appears to induce oxidative stress among career welders [5]. In a study among 43 welders exposed to welding fume containing manganese, 11 cases of manganism were identified presenting with the following symptoms; sleep disturbance, mood changes, bradykinesia, headaches, sexual dysfunction, olfaction loss, muscular rigidity, tremors, hallucinations, slurred speech, postural instability, monotonous voice, and facial masking [24]. Although manganism was observed in highly exposed workers, the scant exposure-response data available for welders do not support a conclusion that welding is associated with clinical neurotoxicity [34].

## 4. Conclusions

This study examined the exposure of welders in industries to welding fume. Results of the investigation showed that mean values of eight hours average particulate concentrations measured during welding at the welders breathing zone were 6.3 mg/m^3^ (Factory 1), 5.3 mg/m^3^ (Factory 2), 11.3 mg/m^3^ (Factory 3), 6.8 mg/m^3^ (Factory 4), 4.7 mg/m^3^ (Factory 5), and 3.0 mg/m^3^ (Factory 6). Mean exposures to welding fumes were above the TLV in Factory 1 (75% confidence), Factory 2 (55% confidence), Factory 3 (97.5% confidence), and Factory 4 (97.5% confidence). Mean exposures were below the TLV in Factory 5 (55% confidence) and Factory 6 (99.99% confidence).

The welders in Factories 1, 2, 3, and 4 were exposed to welding fume concentration above the limit value, which increases the risk of respiratory health problems. Natural air ventilation helped to reduce these levels but the exposures were not considered to be controlled.

Mean values of airborne manganese, copper, and molybdenum levels measured during welding were in the range of 0.010 mg/m^3^–0.477 mg/m^3^, 0.001 mg/m^3^–0.080 mg/m^3^ and 0.001 mg/m^3^–0.058 mg/m^3^, respectively. Measured manganese concentrations in all factories were well below the SASO permissible exposure level (PEL 8h-TWA, ceiling) of 5 mg/m^3^. Measured manganese concentrations in factories 1, 2, 4, and 6 were below the ACGIH threshold limit value (TLV 8h-TWA) of 0.2 mg/m^3^, but they exceeded the limit value in factories 3 and 5 for two samples. Mean values of airborne manganese levels measured during welding were 0.22 mg/m^3^ in Factory 3 and 0.21 mg/m^3^ in Factory 5, which slightly exceed ACGIH threshold limit value (TLV 8h-TWA) of 0.2 mg/m^3^.

Measured copper and molybdenum concentrations in all factories were well below the SASO permissible exposure levels (PEL 8h-TWA) and ACGIH threshold limit values (TLV 8h-TWA). Mean values of calculated equivalent exposures values were: 1.50 (Factory 1), 1.56 (Factory 2), 5.14 (Factory 3), 2.21 (Factory 4), 2.89 (Factory 5), and 1.20 (Factory 6). In all factories equivalent exposure levels were exceeding the threshold limit values, except for one sample taken in Factory 6.

## Figures and Tables

**Table 1.** Sizes of Work Places, Number of Workers and Electrode Types.

**Factory No**	**1**	**2**	**3**	**4**	**5**	**6**
**Workplace size, m** **Volume, m^3^**	64 × 25 × 9 14,400	50 × 25 × 12 15,000	114 × 72 × 12 98,496	40 × 15 × 12 72,000	152 × 45 × 4 27,360	60 × 30 × 10 18,000
**Total Workers**	30	81	100	56	130	19
**Welders**	12	10	35	20	80	6
**Work Schedule (h)** **Break Periods (h)**	07:00–15:00 12:00–13:00	06:00–17:00 09:00–09:15 12:30–13:00 16:00–16.15	07.30–18:00 10:00–10:15 12:45–13:45	07:00–15.30 10:00–10:30 13:00–13:30	07:00–15:30 12:30–13:00	07:00–16:00 9:30–9:45 12:30–13:00 15:00–15:15
**Electrode Type**	E7018	E7024,7018	E7018,7024	E6013	E7024	E7018

**Table 2:** Eight- hour Average Particulate Concentrations of Welding Fume.

**Sample No**	**Particulate Concentration (mg/m^3^)**					

	**Factory 1**	**Factory 2**	**Factory 3**	**Factory 4**	**Factory 5**	**Factory 6**

1	11.0	4.0	13.0	6.0	9.5	3.5
2	15.5	3.0	4.5	6.0	2.0	2.5
3	5.0	10.5	17.0	5.5	4.0	2.5
4	5.0	7.5	4.0	6.0	3.0	3.0
5	5.5	5.0	7.0	4.5	10.5	2.0
6	4.0	5.0	24.0	8.0	13.0	1.5
7	5.0	3.5	3.0	7.0	1.5	4.5
8	8.0	7.0	3.0	12.0	1.0	3.5
9	2.0	4.0	13.5	8.0	1.0	3.5
10	2.0	3.5	24.0	5.0	1.5	3.5

**Table 3:** Eight Hour Average Metal Concentrations of Welding Fume In Each Factory.

**Factory No.**	**Eight hour Average Particulate Concentration (mg/m^3^)**			
	**Total**	**Mn**	**Cu**	**Mo**
1	5.0 5.0 4.0 5.0	0.106 0.116 0.080 0.142	0.002 0.003 0.001 0.004	0.001 0.001 0.001 0.001
2	5.0 5.0 3.5 7.0	0.117 0.062 0.066 0.184	0.002 0.002 0.001 0.006	0.001 0.001 0.001 0.001
3	17.0 24.0 13.5 24.0	0.026 0.477 0.272 0.091	0.001 0.009 0.059 0.080	0.001 0.002 0.001 0.001
4	8.0 7.0 12.0 8.0	0.098 0.085 0.010 0.129	0.002 0.005 0.003 0.003	0.004 0.002 0.001 0.058
5	9.5 4.0 10.5 13.0	0.279 0.088 0.190 0.280	0.022 0.002 0.002 0.003	0.021 0.001 0.001 0.001
6	3.5 2.5 4.5 3.5	0.050 0.187 0.095 0.082	0.002 0.001 0.001 0.002	0.001 0.003 0.003 0.001
